# ANCA-associated vasculitis with isolated splenomegaly as the initial organ presentation

**DOI:** 10.1093/rap/rkae045

**Published:** 2024-03-27

**Authors:** Wataru Kitamura, Masatoshi Kuratsune, Akiko Iseki, Shoichi Kuyama

**Affiliations:** Department of Hematology, National Hospital Organization Iwakuni Clinical Center, Iwakuni, Japan; Department of Hematology, Oncology and Respiratory Medicine, Okayama University Graduate School of Medicine, Dentistry, and Pharmaceutical Sciences, Okayama, Japan; Department of Nephrology, National Hospital Organization Iwakuni Clinical Center, Iwakuni, Japan; Department of Pathology, National Hospital Organization Iwakuni Clinical Center, Iwakuni, Japan; Department of Respiratory Medicine, National Hospital Organization Iwakuni Clinical Center, Iwakuni, Japan

Key message

- Isolated splenomegaly should be recognized as a potential differential diagnosis for ANCA-associated vasculitis.

Dear Editor, Antineutrophil cytoplasmic antibody-associated vasculitis (AAVs) is characterized by necrotizing vasculitis, typically involving small- to medium-sized vessels and showing few or no immune deposits [1]. Although they primarily impact the upper respiratory tract, lungs and kidneys, the disease can progress beyond these organs [2, 3]. Splenic involvement of patients with AAVs, represented by splenomegaly, splenic infarction and splenic rupture, has been recently reported to be possibly more frequent than previously believed [3]. However, to our best knowledge, there are no reports of patients with AAV who had isolated splenomegaly as the initial organ abnormality without the involvement of the upper respiratory tract, lungs or kidneys, which are the preferred sites [1]. Here, we present a patient who had only high-grade continuous fever and splenomegaly at the time of initial presentation, which finally led to the diagnosis of AAVs.

A 64-year-old female presented to our institution with splenomegaly (size: 9.8 × 6.0 × 10.8 cm, volume: 398 cm^3^ [4]) during a follow-up visit following cardiovascular surgery (aortic valve replacement and thoracic aortic aneurysm repair). No mass lesions or infarcts were detected on contrast-enhanced whole-body computed tomography (CT). Her medical history included asymptomatic microscopic haematuria for 20 years (the cause was unknown, as there had been no previous investigation), dyslipidaemia and dizziness. Her only clinical symptom was a high-grade continuous fever lasting a month (38–39°C). Complete blood counts indicated mild anaemia (9.8 g/dl; reference range: 11.6–14.8 g/dl) and thrombocytopenia (105 × 10^3^/μl; reference range: 158–348 × 10^3^/μl) without leukocytopenia. Biochemical tests revealed elevated C-reactive protein (1.59 mg/dl; reference range: 0.00–0.14 mg/dl), lactate dehydrogenase (292 IU/l; reference range: 124–222 IU/l), creatinine (0.82 mg/dl; reference range: 0.46–0.79 mg/dl), and soluble interleukin-2 receptor (2605.0 IU/ml; reference range: 122.0–496.0 IU/ml) with polyclonal hypergammaglobulinemia. *BCR::ABL1* fusion and *JAK2*V617F mutations were absent. Transesophageal echocardiography (TEE) and blood culture showed no abnormalities. Positron emission tomography (PET)-CT revealed mild fluorodeoxyglucose (FDG) uptake (maximum standardized uptake value [SUVmax]: 4.8) only in the spleen (Fig. 1A, arrowheads); uptake in the sternum was considered to be caused by postoperative changes. Primary aggressive splenic lymphoma was suspected initially, but a bone marrow biopsy showed no abnormal lymphoid cells. Further tests indicated elevated proteinase 3-antineutrophil cytoplasmic antibody (PR3-ANCA) (260 IU/ml; reference range: < 3.5 IU/ml) (cytoplasmic-ANCA pattern) and rheumatoid factor (217 IU/ml, reference range: < 15 IU/ml) without elevated anti-cyclic citrullinated peptide antibody and proteinuria. There was no evidence of AAVs in the ear, throat or eye. Skin and renal biopsy specimens showed no evidence of AAVs or intravascular lymphoma. Due to the high risk of haemorrhage, a splenic biopsy was avoided, and the patient was closely monitored and treated with oral antipyretics.

**Figure 1.**
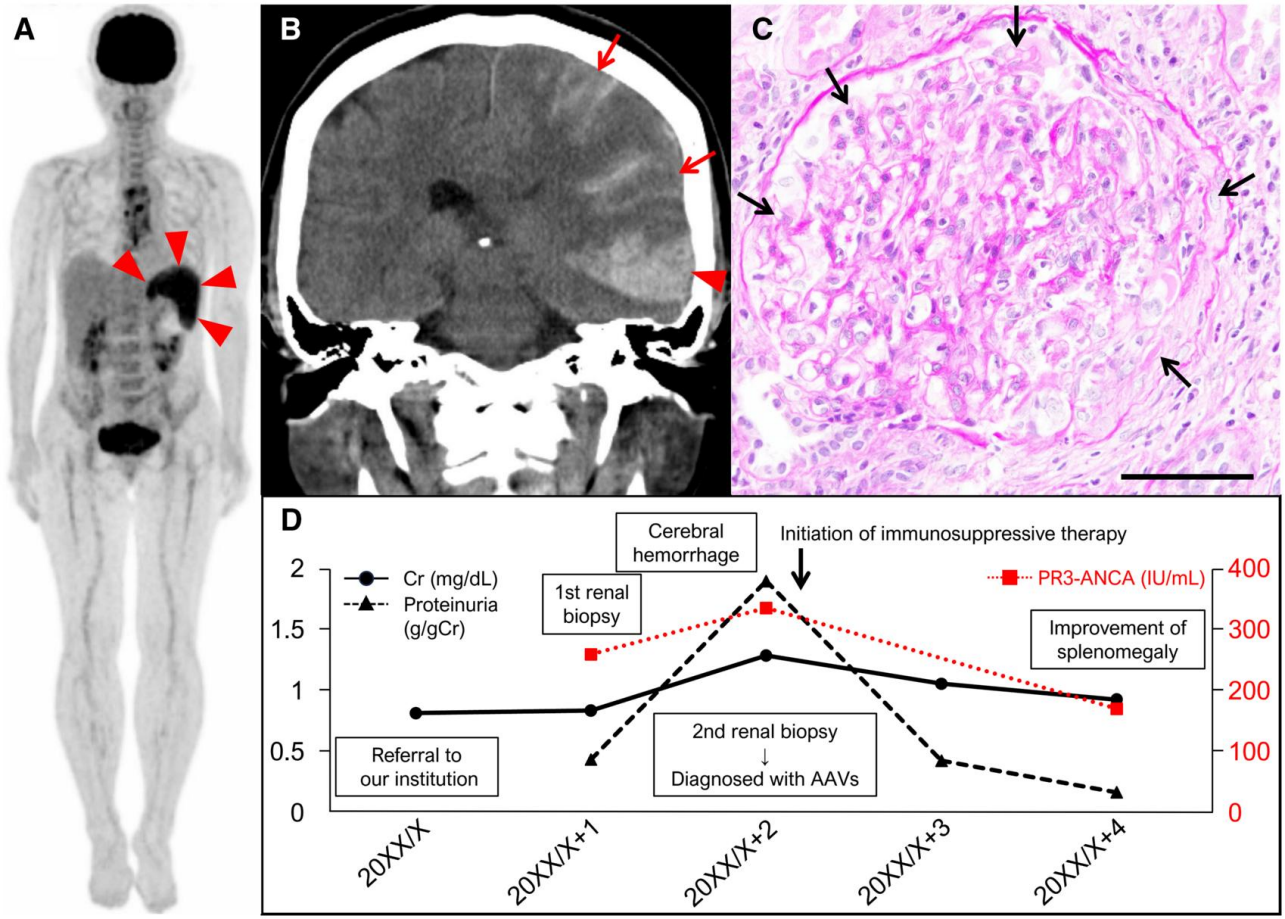
A 64-year-old female with isolated splenomegaly at initial visit, leading to the diagnosis with AAVs. (A) Positron emission tomography-computed tomography (PET-CT) shows an increased uptake of fluorodeoxyglucose (FDG) in the spleen (arrowheads). (B) Brain CT reveals left temporal lobe subcortical (arrowhead) and subarachnoid (arrows) haemorrhages. (C) Periodic acid-Schiff stain of the kidney demonstrates crescentic glomerulonephritis (×200, scale bar: 100 μm; arrows). (D) Laboratory data during clinical course. AAVs: anti-neutrophil cytoplasmic antibody-associated vasculitis; Cr: creatinine; PR3-ANCA: proteinase 3 anti-neutrophil cytoplasmic antibody

Two months post the initial referral, the patient was admitted to our institution with reduced consciousness. Brain CT showed subcortical and subarachnoid haemorrhages in the left temporal lobe (Fig. 1B, arrowhead and arrows). Creatinine levels worsened compared with the initial referral (1.29 mg/dl), and proteinuria emerged (1.90 g/gCr; reference range: < 0.50 g/gCr). Renal biopsy specimens, examined histologically with periodic acid-Schiff staining, revealed crescentic glomerulonephritis (Fig. 1C, arrows). Immunofluorescence analysis showed no immune cell deposits. These findings led to the diagnosis of AAVs. Following immunosuppressive therapy, laboratory data and the patient's neurological status and splenomegaly (size: 9.2 × 4.2 × 7.8 cm, volume: 205 cm^3^ [4]) improved (but anomic aphasia was left) (Fig. 1D).

Various factors, such as infection, haematopoietic tumours, congestion, collagen disease and direct tumour invasion, can cause splenomegaly [5]. Our patient was initially suspected to have a haematopoietic tumour and referred to the Department of Haematology in our institution. However, given the high-grade continuous fever lasting over a month, lack of findings on TEE and bone marrow examination, and reports that splenomegaly resulting from non-malignant conditions generally exhibits a lower SUVmax on PET-CT than that caused by lymphomas (with some exceptions) [6, 7], we considered the possibility of collagen disease including AAVs as a differential diagnosis, despite isolated splenomegaly being an atypical finding. It has been reported that patients with AAVs who required over a year for diagnosis exhibited lower creatinine levels upon admission as observed in our patient [2], suggesting that mild renal findings may have delayed the consideration of AAVs in the differential diagnosis. Since PET-CT has been reported to have the potential to identify occult sites of disease activity in patients with AAVs [8], the isolated mild FDG uptake of the spleen observed in this case was finally considered a lesion caused by AAVs. Our experience highlights the importance of considering AAVs as a differential diagnosis, even when encountering a patient presenting with isolated splenomegaly, accompanied by high-grade continuous fever, and lacking apparent upper respiratory tract, lung or kidney abnormalities.

## Data Availability

The data are available from the corresponding author upon reasonable request.
